# The ESRP1 promoter reporter can function as an in vivo sensor of DNA methyltransferase inhibition

**DOI:** 10.1186/s12896-025-01031-y

**Published:** 2025-08-27

**Authors:** Lecheng Lin, Lingli Chen, Yajie Jing, Zhihong Chen

**Affiliations:** 1 Suzhou Engineering Research Center of Natural Medicine and Functional Food, School of Biological and Food Engineering, Suzhou University, Suzhou, 234000 China; 2 School of Basic Medicine, Youjiang Medical University for Nationalities, Baise, 533000 China

**Keywords:** Epithelial splicing regulatory protein 1 (ESRP1), Promoter reporter, DNA methyltransferase (DNMT), Bioluminescence imaging, Anticancer agents, High-throughput screening (HTS)

## Abstract

**Background:**

The discovery of novel DNA methyltransferase (DNMT) inhibitors as anticancer agents represents a significant milestone in pharmaceutical research. However, the absence of robust high-throughput screening methods for these compounds has substantially hindered their development.

**Results:**

In this study, we found that the epithelial splicing regulatory protein 1 (ESRP1) was underexpressed in renal cell carcinoma (RCC) cells. ESRP1 overexpression induced G1-phase arrest and inhibited the proliferation of RCC cells by downregulating cyclin A2 expression. Furthermore, the ESRP1 promoter was hypermethylated in RCC cells, and treatment with 5-aza-2’-deoxycytidine (5-Aza-CdR), a DNMT inhibitor, effectively demethylated the CpG sites within the promoter region of ESRP1, thereby upregulating the transcriptional activity of the ESRP1 promoter and gene expression both in vitro and in vivo. Additionally, we constructed a bioluminescent reporter gene (designated ESRP1-P-Luc2) by fusing the promoter sequence of the ESRP1 gene with the luciferase gene using molecular cloning techniques. Bioluminescence imaging revealed that 5-Aza-CdR treatment could upregulate the expression of the reporter gene both in vitro and in vivo.

**Conclusions:**

Our results demonstrate that in RCC cells, ESRP1 promoter hypermethylation is accompanied by downregulation of its expression level; restoring ESRP1 expression can induce cell cycle G1-arrest and inhibit RCC cell proliferation by downregulating cyclin A2 expression; ESRP1-P-Luc2 may serve as a useful tool for monitoring the effects of DNMT inhibitor anticancer drugs at both the cellular level and in living animals, thereby providing a potential tool for high-throughput screening (HTS) of such drugs.

**Supplementary Information:**

The online version contains supplementary material available at 10.1186/s12896-025-01031-y.

## Introduction

Epigenetics encompasses the study of heritable changes in gene expression that occur without alterations to the underlying DNA sequence, often driven by environmental factors and behaviors. Among these regulatory mechanisms, DNA methylation is among the most prevalent and well-characterized pathways [1–3].

Critically, dysregulation of epigenetic modifications, particularly aberrant DNA methylation, is increasingly recognized as a major driver of tumorigenesis [4]. Central to this process is the hypermethylation of tumor suppressor gene promoters, which induces their transcriptional silencing and thereby promotes cancer development [5, 6]. Therapeutically, DNMT inhibitors target this mechanism by reversing promoter hypermethylation, thereby reactivating silenced tumor suppressor genes and positioning them as promising anticancer agents [7–9]. Consequently, major pharmaceutical companies are actively pursuing the development of novel DNMT inhibitors for cancer therapy [10]. However, a critical bottleneck hindering this development is the lack of robust high-throughput screening (HTS) tools specifically designed to detect DNA methylation-dependent gene reactivation.

Bioluminescence imaging (BLI), which uses luciferase reporter genes, has emerged as a powerful technology for noninvasively and dynamically monitoring cellular processes, such as gene expression and drug responses, both in vitro and in vivo. This real-time, noninvasive capability makes BLI particularly valuable for applications in drug screening and therapeutic efficacy evaluation [11, 12]. Reporter gene systems for BLI can be broadly categorized into two types: first, those incorporating transcriptional regulatory regions (e.g., promoters, enhancers) to report transcriptional activity; second, those utilizing fusion proteins between the gene of interest and luciferase, which are used for studying protein interactions, localization, or degradation [13]. Given that DNMT inhibitors exert their anticancer effects primarily by demethylating specific promoter regions and consequently upregulating the transcription of silenced tumor suppressor genes, promoter-driven luciferase reporter systems are an ideal approach for screening these compounds. Therefore, the identification of a tumor suppressor gene silenced by promoter hypermethylation in a specific cancer type provides a rational basis for constructing such a promoter-driven BLI reporter system for the DNMT inhibitor HTS.

Epithelial splicing regulatory protein 1 (ESRP1), an epithelial cell-specific splicing factor, regulates posttranscriptional gene expression through alternative splicing, influencing cellular behavior [14–16]. It is implicated in tumorigenesis and progression, with evidence suggesting that it often acts as a tumor suppressor; however, several studies have reported its pro-oncogenic role in specific cancer contexts [17–19]. In this context, this study first investigated ESRP1 expression and promoter methylation status in renal cell carcinoma (RCC) cells, exploring its inhibitory role in RCC proliferation and the underlying mechanisms. Consistent with the potential tumor suppressor role of ESRP1 observed in this study, we found that ESRP1 expression is suppressed by promoter hypermethylation in RCC. On the basis of this discovery, we designed and validated a bioluminescent reporter system driven by the ESRP1 promoter. We then demonstrated the utility of this reporter for real-time monitoring of the activity of the demethylating agent 5-aza-2’-deoxycytidine (5-Aza-CdR) both in vitro and in vivo. The primary goal of this work was to establish an effective HTS tool to facilitate the discovery of novel DNMT inhibitor-based anti-renal cancer drugs.

## Materials and methods

### Bioinformatics analysis methods

ESRP1 mRNA and protein expression levels in carcinoma and normal tissues were determined via the UALCAN database (http://ualcan.path.uab.edu/). Immunohistochemical (IHC) staining of ESRP1 in kidney renal clear cell carcinoma **(**KIRC) and normal tissues was performed via the Human Protein Atlas (HPA) database (https://www.proteinatlas.org/). The expression levels of ESRP1 in different RCC cell lines were analyzed via data from the Cancer Cell Line Encyclopedia (CCLE) database (https://sites.broadinstitute.org/ccle/).

### Cell culture, cell transfection, and stable cell line screening

A498 and HEK293 cells were purchased from Wuhan Pu-nuo-sai Life Technology Co. Ltd. (Wuhan, China). The cells were cultured in MEM supplemented with 10% fetal bovine serum (FBS) at 37 °C in a 5% CO₂ atmosphere. siRNAs were synthesized by GenePharma Co., Ltd. (Shanghai, China): human cyclin A2, sense: 5′-GGAUCUUCCUGUAAAUGAUTT-3′, antisense: 5′-AUCAUUUACAGGAAGAUCCTT-3′; control scramble siRNA, sense: 5′-UUCUCCGAACGUGUCACGUTT-3′, antisense: 5′-ACGUGACACGUUCGGAGAATT-3′. The pcDNA3.1-Luc2 and pGL4.19-ESRP1-P-Luc2 vectors were purchased from the Public Protein/Plasmid Library (Nanjing, China). For transfections, A498 cells were seeded at 2 × 10⁵ cells per well in 6-well plates, allowed to adhere overnight, and then transfected with plasmids or siRNAs via HighGene transfection reagent (ABclonal, Wuhan, China) according to the manufacturer’s protocol. To establish stable A498-Luc2 and A498-ESRP1-P-Luc2 cell lines, the cells were transfected with the respective plasmids. At 24 h post-transfection, the medium was removed, and the cells were washed twice with prewarmed (37 °C) PBS and then cultured in MEM containing 10% FBS and 800 µg/mL G418. The G418 selection medium was replaced every 2–3 days until distinct resistant colonies became clearly visible. The cells were digested with 0.25% trypsin and transferred to new culture dishes for expansion.

### Bisulfite sequencing PCR (BSP) and methylation-sensitive restriction enzyme-qPCR (MSRE-qPCR)

Bisulfite sequencing PCR (BSP) was performed by Sangon Biotech Co., Ltd. (Shanghai, China). For methylation-sensitive restriction enzyme-qPCR (MSRE-qPCR), genomic DNA was extracted and purified. To assess ESRP1 promoter methylation status, DNA samples (1 µg) were digested with the methylation-sensitive enzyme HhaⅠ (12 h) (New England Biolabs, Beijing, China) in a 20 µL reaction volume. Digestion was performed using 1 µL of HhaⅠ at 37 °C. MSRE-qPCR analysis was performed using primers specifically targeting an ESRP1 promoter fragment containing an HhaⅠ recognition site within CpG Island 1 (forward: 5′-GCTCCATTGTGCCTGAGTTTCC-3′, reverse: 5′-CACGCTCGTCCCAAACCCG-3′; synthesized by Sangon Biotech) and primers targeting a known unmethylated internal reference genomic locus (forward: 5′-AGGCAAATAAAAGGAAATGGG-3′, reverse: 5′-GCAAGGAAACTCAGGCACAAT-3′; synthesized by Sangon Biotech). qPCR was performed via a LightCycler 96 system (Roche, Germany) with SYBR Green Master Mix. The reaction conditions were as follows: initial denaturation at 95 °C for 5 min; 40 cycles of denaturation at 95 °C for 10 s; and combined annealing/extension at 60 °C for 40 s. Data were analyzed via the 2^−ΔΔCT^ method.

### RNA isolation, cDNA synthesis, and quantitative real-time PCR (RT-qPCR)

Total RNA was isolated from cell lines or tissues using the Quick-RNA Miniprep Kit (Zymo Research, USA) according to the manufacturer’s instructions. The RNA concentration and purity were determined spectrophotometrically (A260/A280 ratio). First-strand cDNA was synthesized from 1 µg of total RNA using the All-in-One First-Strand cDNA Synthesis Kit (GeneCopoeia, China) following the provided protocol. The mRNA expression levels of ESRP1 and the internal control gene GAPDH were quantified by RT-qPCR using gene-specific primers (human ESRP1: HSQRP013766; human GAPDH: HSQRP20026; supplied by GeneCopoeia Inc.). qPCR amplification and detection were performed using the same procedure as described in Section “Bisulfite sequencing PCR (BSP) and methylation-sensitive restriction enzyme-qPCR (MSRE-qPCR)”.

### Soft agar colony assay

A498 cells were seeded at a density of 2 × 10⁵ cells per well in 6-well plates and allowed to adhere overnight under standard culture conditions (37 °C, 5% CO₂). After adhesion, cells were transfected with 4 µg of either the ESRP1 expression plasmid (pcDEF3-ESRP1) or the empty vector control (pcDEF3) using HighGene transfection reagent according to the manufacturer’s protocol. At 24 h post-transfection, cells were harvested by trypsinization, counted, and resuspended in complete medium. For the soft agar assay, a two-layer system was prepared. A 1.5 mL base layer of 0.6% Select Agar in complete medium was solidified per well in new 6-well plates. The cells (5000 cells/well) were then resuspended in 1.5 mL of 0.3% Select Agar in complete medium (preequilibrated at 40 °C) and immediately layered on top of the preset base layer. The plates were incubated undisturbed at 37 °C with 5% CO₂ for 3 weeks; fresh medium (1 mL) was added carefully to the edge of each well weekly to prevent drying. Colonies (> 50 cells) were counted and photographed using phase-contrast optics on an inverted microscope.

### Cell proliferation, cell cycle analysis, and luciferase activity analysis

Bioluminescence imaging (BLI) was used to assess cell proliferation. A498 cells were seeded in 96-well plates at a density of 1 × 10⁴ cells/well and allowed to adhere overnight at 37 °C with 5% CO₂. The cells were then either transfected with pcDEF3-ESRP1 (0, 0.1, and 0.3 µg) using HighGene reagent, or treated with 5-Aza-CdR (0, 1, 2, and 5 µM). After 48 h of treatment, D-Luciferin was added to the culture medium (150 µg/mL), and after 5 min incubation, photon emission was quantified using an IVIS Lumina LT; photon emission was measured within tumor regions and quantified using system software. Cell cycle analysis and luciferase activity analysis were performed as described previously [13].

### Western blotting assay and immunohistochemical analyses

Western blotting and immunohistochemistry (IHC) were performed as previously described [20]. The primary antibodies used included anti-β-actin (Abcam, UK), anti-vinculin (ABclonal, China), anti-cyclin A2 (ABclonal, China), anti-Ki67 (Abcam, UK), and anti-ESRP1 (Abcam, UK) antibodies.

### Tumor xenografts

The animal experiments followed the Guide for the Care and Use of Laboratory Animals (National Academies Press, USA). Female severe combined immunodeficiency (SCID) mice and BALB/c nude mice (4–5 weeks old) were obtained from Beijing Weitong Lihua Experimental Animal Technology Co., Ltd. (Beijing, China). A498-ESRP1-P-Luc2 cells (1 × 10⁷ in 0.2 mL of PBS) were injected subcutaneously into both flanks of anesthetized SCID mice. Posttumor formation, the tissues were excised, dissected into approximately 1–2 mm³ blocks, and subcutaneously inoculated into BALB/c nude mice. When the tumors reached approximately 100 mm³ in size, the mice were randomized into two groups (*n* = 3‒4/group). Adeno-associated virus (AAV) was obtained from Hanbio Biotechnology Co., Ltd. (Shanghai, China). For AAV delivery, AAV/ESRP1 or AAV/Null was injected into tumors twice weekly, while tumor formation was monitored three times per week throughout the 19-day experimental period. For 5-Aza-CdR treatment, the mice received intraperitoneal injections of 5-Aza-CdR (2 mg/kg) or vehicle solution (PBS containing an equivalent concentration of DMSO) three times throughout the same 19-day period. The mice were then sacrificed for xenograft weight measurement.

### In vivo bioluminescen imaging and luciferase assay

For in vivo bioluminescence imaging, mice were divided into two groups: the experimental group (*n* = 9), in which each nude mouse was intraperitoneally injected with 2 mg/kg 5-Aza-CdR, and the control group (*n* = 9), in which each nude mouse received the vehicle solution (PBS containing an equivalent concentration of DMSO). At 48 h after 5-Aza-CdR or vehicle treatment, all mice were intraperitoneally injected with D-luciferin (150 mg/kg). Tumor bioluminescence was acquired using an IVIS Lumina LT imaging system, and photon flux within tumor regions was quantified using Living Image software (PerkinElmer).

### Statistical analysis

Statistical analysis was performed via GraphPad Prism 9.5. The data are presented as the means ± SEMs. Welch’s t test or paired t test was used for two-group comparisons, while multiple comparisons were analyzed by Welch’s t test with Bonferroni correction, and significance was defined as *p* < 0.05.

## Results

### ESRP1 was underexpressed in RCC cells and exhibited promoter region hypermethylation

**Fig. 1:**
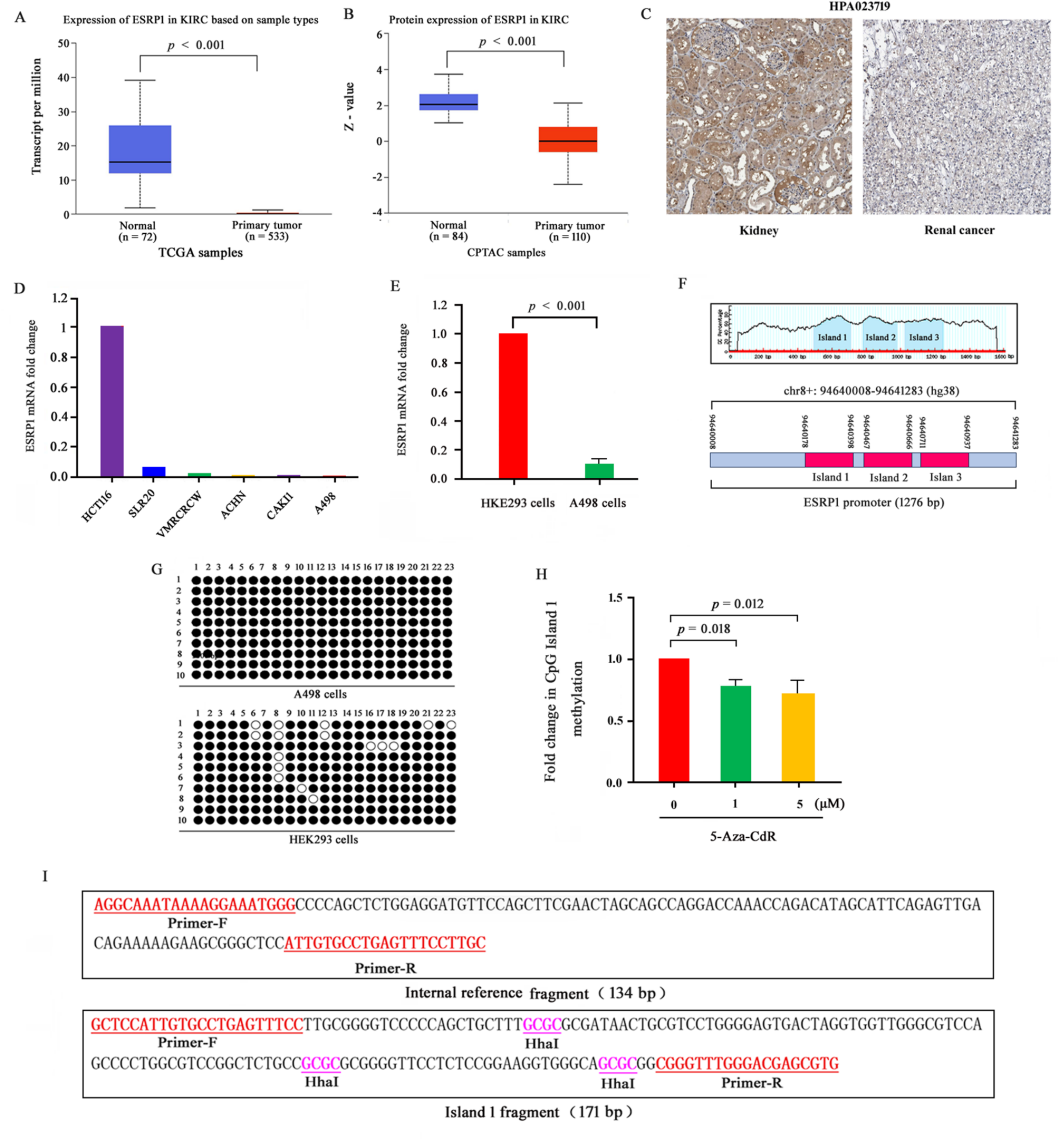
Expression and promoter methylation status of ESRP1 in RCC cells. (**A**, **B**) ESRP1 mRNA (**A**) and protein (**B**) expression in KIRC carcinoma tissue and paracarcinoma tissue from the UALCAN database. (**C**) ESRP1 expression in KIRC carcinoma tissue and paracarcinoma tissue from the HPA database. (**D**) ESRP1 mRNA expression in various RCC cell lines from the CCLE database. (**E**) The expression of ESRP1 in A498 and HEK293 cells was analyzed by RT-qPCR. (**F**) Analysis of CpG Island distribution within the ESRR1 promoter region (1276 bp) using MethPrimer (top); precise length and sequence coordinates of the ESRP1 promoter region (bottom). (**G**) Bisulfite sequencing of CpG Island 1 in the ESRP1 promoter region was performed in HEK293 and A498 cell lines. Filled circles represent methylated CpG sites within the ESRP1 CpG Island 1, and open circles denote unmethylated CpG sites. (**H**) Island 1 and internal reference fragment DNA sequences. (**I**) A498 cells were treated with 0, 1, and 5 µM 5-Aza-CdR for 48 h. The methylation level of CpG Island 1 in the ESRP1 promoter was then assessed by methylation-sensitive restriction enzyme-qPCR (MSRE-qPCR). For this analysis, genomic DNA from A498 cells was digested with the HhaI for 12 h at 37 °C. Data are from three independent experiments. Statistical significance in Fig. 1**E** was assessed using Welch’s t-tests, while corrections for multiple comparisons were applied to Fig. 1**H**. Error bars represent standard error, and exact *p*-values are shown above comparison bars

UALCAN and HPA databases were used to analyze ESRP1 expression levels in KIRC tissues. Compared to normal tissues, KIRC tissues showed significantly reduced ESRP1 mRNA and protein levels (Fig. 1A-C). We further utilized the CCLE database to examine ESRP1 expression in RCC cell lines, including SLR20, VMRCRCW, ACHN, CAKI1, and A498, by using the HCT116 cell line as a control which showed high ESRP1 expression according to CCLE data. ESRP1 expression was significantly lower in RCC cell lines than in HCT116 cells, and A498 cells presented the lowest expression (Fig. 1D). Next, RT-qPCR was performed to assess the ESRP1 expression level in A498 and human embryonic kidney 293 (HEK293) cells. The results showed that the expression level of ESRP1 in A498 cells was significantly lower than that in HEK293 cells (Fig. 1E). Given that promoter methylation is a key regulatory mechanism of gene downregulation, we analyzed the methylation status of the ESRP1 promoter in A498 cells and HEK293 using bisulfite sequencing PCR (BSP). MethPrimer online tool analysis revealed that ESRP1 CpG Island 1 contains a total of 23 CpG sites. BSP results indicated that the methylation rate of these sites reached 100% in the renal cancer cell line A498. In contrast, HEK293 cells showed an overall methylation rate of 91% across the 23 sites, which is lower than that in the renal cancer cells, with only the 8th site exhibiting a methylation rate of 50% (Fig. 1F and G). Next, the effect of 48 h treatment with the DNMT inhibitor 5-aza-2’-deoxycytidine (5-Aza-CdR) on the methylation level of CpG Island 1 was evaluated using MSRE-qPCR. As expected, 5-Aza-CdR treatment significantly reduced the methylation level of CpG Island 1 (Fig. 1H and I). These data revealed the presence of hypermethylated CpG sites within CpG Island 1 of the ESRP1 promoter, and suggested that the low expression of ESRP1 in KIRC is associated with this hypermethylation.

### Restoration of ESRP1 expression suppressed the tumorigenic potential and proliferation of RCC cells through cell cycle arrest induction in the G1-phase

**Fig. 2:**
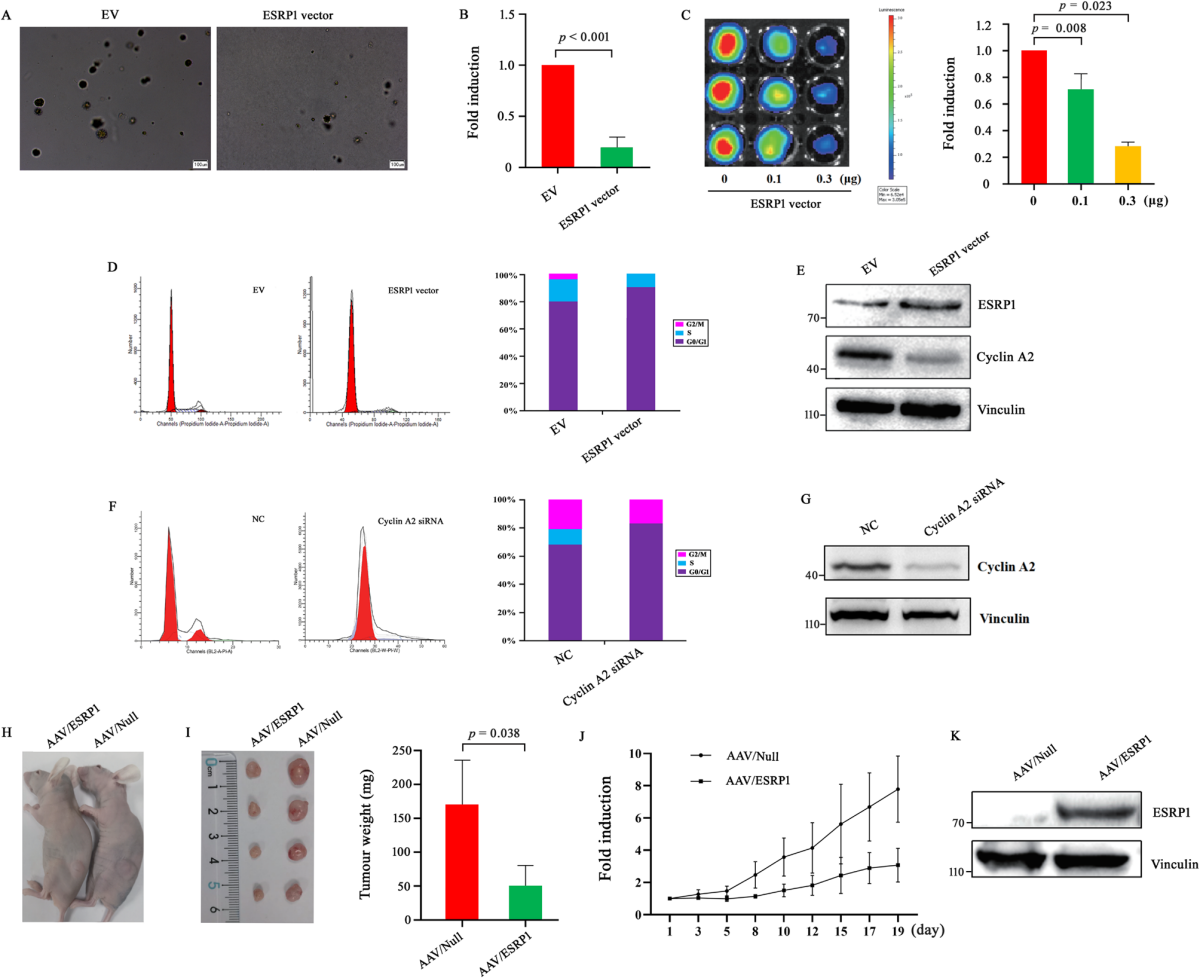
ESRP1 overexpression inhibits tumorigenicity and proliferation of A498 cells and induces G1-phase arrest. (**A**, **B**) The effect of ESRP1 overexpression on the colony-forming ability of A498 cells was analyzed using a soft agar assay. Colonies were imaged and counted after 3 weeks; representative images were shown in (**A**). The number of colonies formed by control cells transfected with the empty vector (EV) was set to 1 for normalization (**B**). (**C**) A498-Luc2 cells transfected with ESRP1 vector were imaged by IVIS Lumina LT system. Representative bioluminescence images (left) and total flux quantification (right) are shown. (**D**) Flow cytometry analysis using propidium iodide (PI) staining was performed to assess cell cycle distribution in A498 cells 48 h after transfection with the ESRP1 vector or the empty vector (EV) control. (**E**) Western blotting analysis of ESRP1 and cyclin A2 protein levels in A498 cells was performed following transfection with the ESRP1 expression vector or EV. (**F**) Flow cytometry analysis using propidium iodide (PI) staining was performed to assess cell cycle distribution in A498 cells 48 h after transfection with 50 nM cyclin A2 siRNA or negative control siRNA (NC). (**G**) Western blotting analysis of cyclin A2 protein levels in A498 cells was performed following cyclin A2 siRNA or NC treatment. (**H**-**K**) Nude mice bearing A498 cell xenografts were administered intratumoral injections of adeno-associated virus (AAV)-ESRP1 or AAV-Null (control). Representative images of xenografts were shown in (**H**). Tumor weights (**I**) and tumor volume growth curves (**J**) were presented. ESRP1 protein expression in tumor tissues was analyzed by Western blotting (**K**). Data are from three independent experiments. Statistical significance in Fig. 2**B** and **I** was assessed using Welch’s t-tests, while corrections for multiple comparisons were applied to Figs. 2**C**. Error bars represent standard error, and exact *p*-values are shown above comparison bars

To determine whether ESRP1 influences RCC, we performed in vitro and in vivo experiments. First, soft agar colony-forming assays were conducted to assess the effect of ESRP1 overexpression on the colony-forming ability of A498 cells. Compared with empty vector (EV)-transfected cells, ESRP1 vector-transfected cells presented a reduced number of clones (Fig. 2A and B). Next, bioluminescence imaging (BLI) was utilized to examine the effect of ESRP1 overexpression on A498 cell proliferation. Either ESRP1 or EV was used to transfect A498 cells expressing luciferase, and changes in cell bioluminescence signal intensity were observed as a cell count indicator. A substantially reduced signal intensity was observed in ESRP1-overexpressing A498 cells compared with EV controls, indicating suppressed cell proliferation (Fig. 2C). Moreover, flow cytometry revealed that ESRP1 overexpression significantly increased the number of cells at the G1 checkpoint as compared to the control group; the Western blotting assay demonstrated that ESRP1 vector-transfected cells exhibited a significant reduction in cyclin A2 protein expression compared to control cells. (Figure 2D and E). Previous research has indicated that suppressing cyclin A2 expression results in G1-phase arrest [21]. Consistent with these findings, the present study revealed that treatment with cyclin A2 siRNA induced G1-phase arrest in A498 cells (Fig. 2F and G). These results suggested that ESRP1-induced downregulation of cyclin A2 expression might induce cell cycle arrest in the G1-phase in human RCC cells. To confirm the in vitro findings shown above, an in vivo xenograft RCC nude mouse model was established. Once tumors reached a palpable size, adeno-associated virus (AAV)/ESRP1 or AAV/Null was injected intratumorally, and AAV/ESRP1 significantly inhibited RCC tumor growth in mouse models (Fig. 2H-K). Collectively, these findings indicated that ESRP1 suppressed the tumorigenic ability of RCC cells by repressing cyclin A2 both in vitro and in vivo.

### 5-Aza-CdR treatment restored the expression of ESRP1 in RCC cells in *vitro* and in vivo

**Fig. 3:**
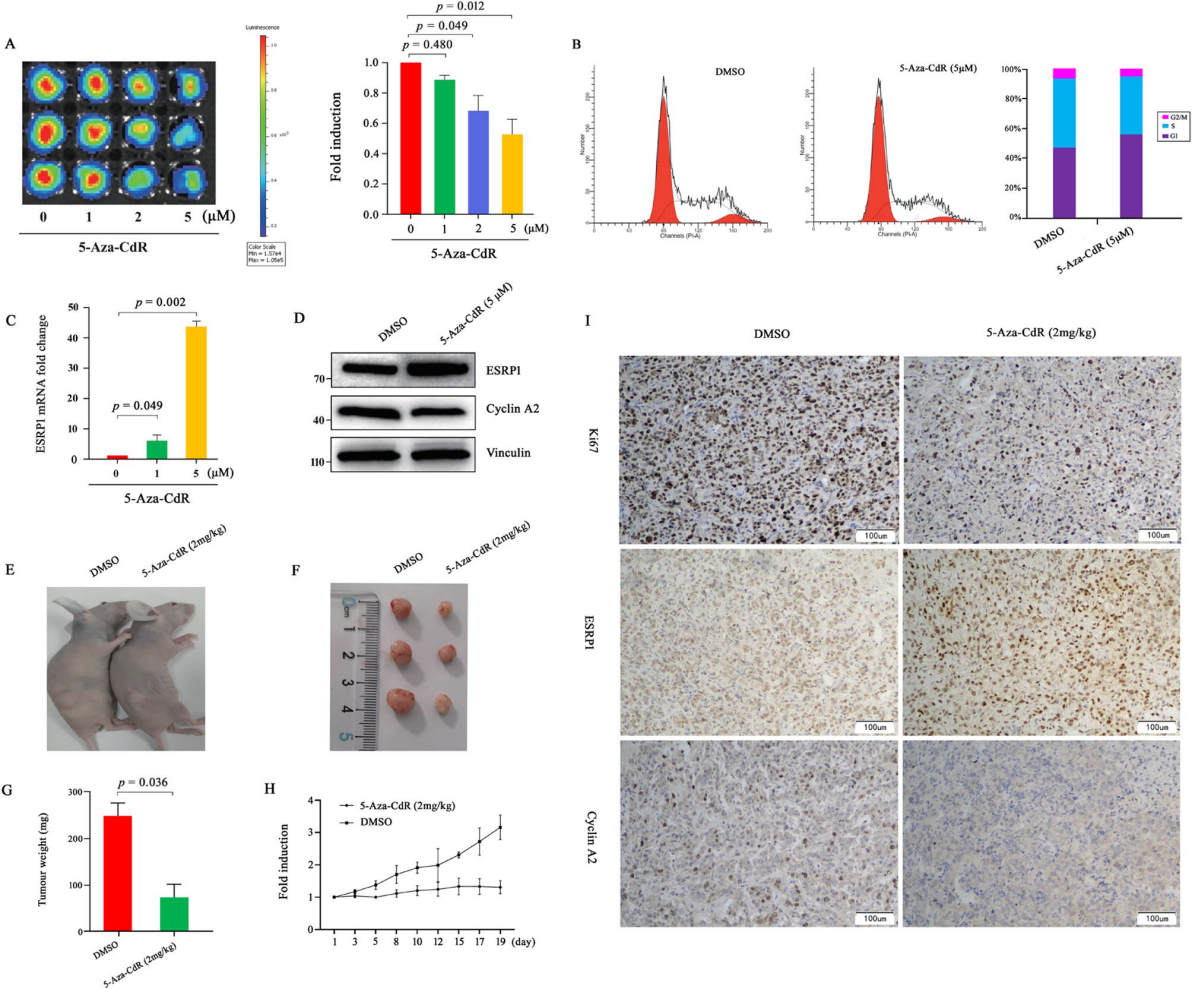
5-Aza-CdR upregulates ESRP1 expression and affects tumor growth in A498 cells. (**A**) A498-Luc2 cells were treated with 0, 1, 2, and 5 µM 5-Aza-CdR for 48 h, and imaged using the IVIS Lumina LT system to obtain FLUX measurements. Representative bioluminescence images (left) and quantification of total flux (right) are shown. (**B**) The cell cycle distribution of A498 cells treated with 5 µM 5-Aza-CdR or DMSO control for 48 h was analyzed by flow cytometry using propidium iodide (PI) staining. (**C**, **D**) ESRP1 expression levels in A498 cells treated with 5 µM 5-Aza-CdR or DMSO control for 48 h were detected by RT-qPCR (**C**) and Western blotting (**D**). (**E**-**G**) Following treatment with 5-Aza-CdR or vehicle solution, the tumors were excised and analyzed. Representative images of tumor-bearing nude mice (**E**), excised tumors from each group (**F**), and statistical analysis of tumor weights (**G**) are shown. (**H**) Tumor volumes were measured three times per week during the treatment period, and growth curves were plotted. (**I**) Immunohistochemical staining of ESRP1, cyclin A2, and Ki67 in representative tumor sections from control and 5-Aza-CdR-treated mice. Data are from three independent experiments. Statistical significance in Fig. 3**A** was assessed using Welch’s t-test with correction for multiple comparisons, while Fig. 3**G** was analyzed without correction. Error bars represent standard error, and exact *p*-values are shown above comparison bars

Our data showed that hypermethylation of CpG sites in the ESRP1 promoter occurred in RCC cells, and ESRP1 overexpression inhibited RCC cell proliferation. Since the DNA methyltransferase inhibitor 5-Aza-CdR can reactivate tumor suppressor genes silenced by promoter methylation, we examined whether it could upregulate ESRP1 expression in RCC cells. First, we confirmed the drug’s efficacy by analyzing its inhibitory effects in vitro. Cell proliferation and flow cytometry assays demonstrated that 5-Aza-CdR significantly suppressed RCC cell proliferation by inducing G1-phase arrest (Fig. 3A and B). Subsequently, RT-qPCR and Western blotting revealed that 5-Aza-CdR increased ESRP1 expression at both the mRNA (Fig. 3C) and protein (Fig. 3D) levels. To validate these findings in vivo, we performed xenograft experiments in nude mice. 5-Aza-CdR treatment significantly reduced tumor weight and volume compared to controls (Fig. 3E–H). Immunohistochemical (IHC) analysis further showed elevated ESRP1 expression accompanied by decreased levels of the proliferation marker Ki67 and cyclin A2 in treated tumors (Fig. 3I). Together, these results demonstrate that 5-Aza-CdR upregulates ESRP1 and exerts antitumor effects in both in vitro and in vivo models.

### Response of reporter cells to 5-Aza-CdR treatment

**Fig. 4:**
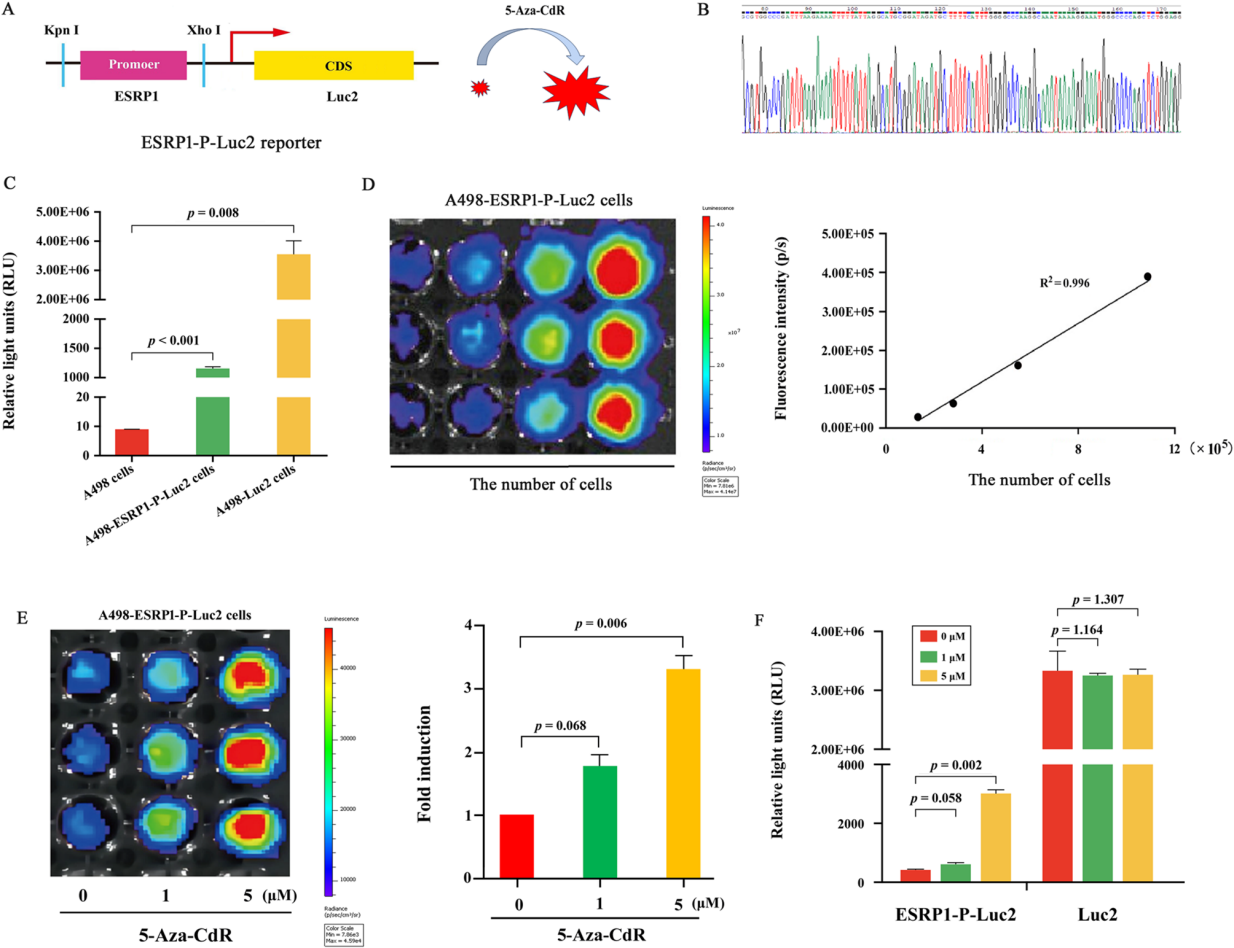
In vitro monitoring of 5-Aza-CdR effects using the ESRP1-P-Luc2 reporter in A498 cells. (**A**) Schematic diagrams of the ESRP1-P-Luc2 vectors used in this study. (**B**) ESRP1-P-Luc2 vectors were constructed and verified by sequencing. (**C**) A498 parental cells and engineered variants (A498-Luc2, A498-ESRP1-P-Luc2) were seeded in 24-well plates (4 × 10⁴ cells/well). Following 48 h of culture, cells were harvested in passive lysis buffer. Luciferase activity in lysates was quantified using a luminometer and normalized to protein concentration. (**D**) A498-ESRP1-P-Luc2 cells were serially diluted, seeded onto a 96-well plate, and immediately imaged using the IVIS Lumina LT system to obtain FLUX measurements (left). These data were averaged and used to generate a plot comparing total flux to cell number (right). (**E**) A498-ESRP1-P-Luc2 cells were placed into wells of a 96-well plate (1 × 10⁴ cells/well). Images were obtained after 48 h of treatment with 5-Aza-CdR (0, 1, and 5 µM). Representative bioluminescence images (left) and quantification of total flux (right) are shown. (**F**) After treatment with 5-Aza-CdR (0, 1, and 5 µM) for 48 h, A498-Luc2 cells and A498-ESRP1-P-Luc2 cells were lysed, and cell lysates were assayed for luciferase activity. Data are from three independent experiments. Statistical significance in Fig. 4**C** was assessed using Welch’s t-tests, while corrections for multiple comparisons were applied to Fig. 4**E** and **F**. Error bars represent standard error, and exact *p*-values are shown above comparison bars

To establish a monitoring tool for evaluating the efficacy of DNMT inhibitor class anticancer agents, we constructed a luciferase reporter plasmid, pGL4.19-ESRP1-P-Luc2, driven by the ESRP1 promoter (Fig. 4A and B). This plasmid or the positive control plasmid pcDNA3.1-Luc2 (driven by the strong constitutive CMV promoter) was transfected into A498 cells. Following the selection process detailed in materials and methods, stably transfected A498-ESRP1-P-Luc2 and A498-Luc2 cell lines were established. Luciferase activity assays conducted in A498-ESRP1-P-Luc2 cells confirmed reporter expression. Results demonstrated a 140-fold increase in luciferase activity in A498-ESRP1-P-Luc2 cells compared to untransfected A498 controls. As expected, the A498-Luc2 control cells exhibited a substantially higher, 394,460-fold increase (Fig. 4C). These data validate the successful generation of both stable reporter cell lines A498-ESRP1-P-Luc2 and A498-Luc2. A498-ESRP1-P-Luc2 cells were subsequently treated with the DNMT inhibitor 5-Aza-CdR. Luciferase activity analysis and bioluminescence imaging performed at 48 h post-treatment revealed that 5-Aza-CdR induced a significant upregulation of luciferase activity and an increase in bioluminescence signal intensity (Fig. 4E and F). These findings indicate that the ESRP1-P-Luc2 reporter system effectively reflects the cellular effects of DNMT inhibitors, such as 5-Aza-CdR, at the in vitro level.

### Imaging in vivo pharmacodynamics of 5-Aza-CdR

**Fig. 5:**
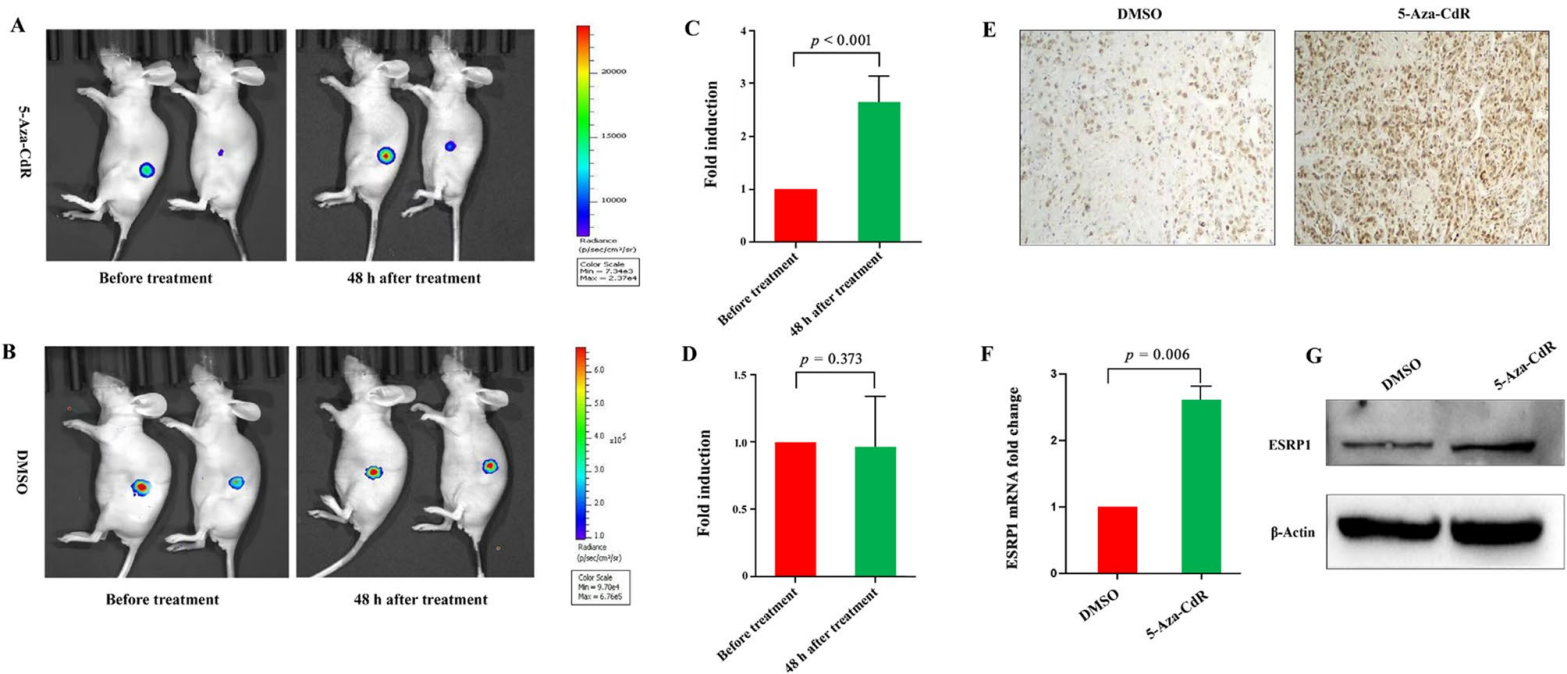
In vivo monitoring of 5-Aza-CdR effects using the ESRP1-P-Luc2 reporter in A498 xenografts. (**A**-**D**) In A498-ESRP1-P-Luc2 tumor-bearing nude mice, bioluminescence imaging and analysis were performed 48 h post-intraperitoneal 5-Aza-CdR (2 mg/kg) administration or vehicle solution. (**E**) Tumor samples at 48 h after 5-Aza-CdR or vehicle solution treatment were immunohistochemically stained with ESRP1 antibodies (magnification: ×200). (**F**, **G**) Following treatment with 5-Aza-CdR or vehicle solution, the mRNA (**F**) or protein (**G**) expression levels of ESRP1 were determined using RT-qPCR or Western blotting analysis. Data are from three independent experiments. Statistical significance in Fig. 5**C** and **D** was evaluated using paired t-tests, while Welch’s t-test was applied for Fig. 5**F**. Error bars represent standard error, and exact *p*-values are shown above comparison bars

To further verify the above findings in vivo, we assessed the effect of 5-Aza-CdR on tumor bioluminescence signal intensity using a mouse subcutaneous RCC model. Since A498 cells exhibit low tumorigenicity upon direct subcutaneous injection in BALB/c nude mice, we employed a tissue passage method incorporating Severe Combined Immunodeficiency (SCID) mice. Briefly, A498-ESRP1-Luc2 or A498-Luc2 cells were first injected subcutaneously into immunodeficient SCID mice (which possess higher tumorigenicity**)**, leading to palpable tumor formation after approximately 4 weeks. The resulting tumor tissues were then resected from these SCID donor mice, minced into small fragments, and subcutaneously implanted into BALB/c nude mice to establish the bioluminescence RCC model in this strain. After a 3-week inoculation period, tumor-bearing BALB/c nude mice were randomly assigned to experimental and control groups. The experimental group received 2 mg/kg 5-Aza-CdR intraperitoneally, while the control group received the vehicle solution. In vivo BLI was performed before and 48 h after treatment. In the experimental group, 5-Aza-CdR treatment resulted in a significant 2.6-fold increase in tumor bioluminescence signal, whereas the control group receiving vehicle solution showed no significant change (Fig. 5A-D). Subsequently, tumor tissues were harvested for further analysis. IHC, RT-qPCR, and Western blotting analyses confirmed that 5-Aza-CdR treatment significantly upregulated ESRP1 expression at both the mRNA and protein levels compared to the control group (Fig. 5E-G). Collectively, these data indicate that the observed increase in bioluminescence signal corresponded with ESRP1 upregulation, confirming the responsiveness of the ESRP1 promoter. Consequently, the ESRP1-P-Luc2 reporter system proved to be a reliable tool for noninvasively monitoring the pharmacodynamic effects of 5-Aza-CdR treatment, specifically its activation of the ESRP1 promoter, in this subcutaneous RCC model.

## Discussion

Our findings confirmed the tumor-suppressive role of ESRP1 in RCC. We demonstrated that ESRP1 overexpression inhibited RCC cell proliferation and induced G1-phase arrest via cyclin A2 downregulation. These findings mechanistically align with our previous observations in cervical cancer, where ESRP1 promoted the degradation of cyclin A2 mRNA to trigger G1 arrest [20]. Collectively, our data establish the ESRP1/cyclin A2 axis as an evolutionarily conserved machinery governing G1-phase arrest in diverse malignancies. Furthermore, we elucidated the epigenetic mechanism underlying ESRP1 downregulation in RCC. Reduced ESRP1 expression was associated with promoter hypermethylation, whereas treatment with the DNMT inhibitor 5-Aza-CdR specifically demethylated CpG sites in the ESRP1 promoter. This finding aligns with the well-established paradigm that promoter hypermethylation silences tumor suppressor genes in cancer and is particularly consistent with previous reports linking ESRP1 downregulation to promoter hypermethylation in tumors [22–24]. Thus, we conclude that promoter hypermethylation is a key mechanism suppressing ESRP1 in RCC, and 5-Aza-CdR reactivates ESRP1 primarily through promoter demethylation.

Building upon the epigenetic responsiveness of the ESRP1 promoter, we developed a novel bioluminescent reporter system for screening DNMT inhibitors against RCC. This system utilizes a luciferase reporter vector driven by the ESRP1 promoter, stably integrated into A498 cells to generate the A498-ESRP1-P-Luc2 cell line. The selection of A498 cells was informed by prior evidence demonstrating their responsiveness to 5-Aza-CdR-mediated demethylation and reactivation of tumor suppressor genes (e.g., WNT7A, KRT19, BTG3) [25, 26], confirming sufficient endogenous DNMT activity for pharmacological inhibition. As hypothesized, 5-Aza-CdR treatment significantly enhanced bioluminescence intensity in A498-ESRP1-P-Luc2 cells, concomitant with reduced ESRP1 promoter methylation and upregulated endogenous ESRP1 expression. A robust inverse correlation was observed between promoter methylation levels and both luciferase activity (Pearson’s *r* = − 0.8311, *p* = 0.0055) and bioluminescence signal intensity (Pearson’s *r* = − 0.744, *p* = 0.022), further supporting methylation-dependent transcriptional reactivation. These results validated the system’s capacity to specifically detect DNMT inhibitor activity via ESRP1 promoter demethylation and subsequent reporter activation.

The development of effective HTS tools is paramount for advancing novel DNMT inhibitors, particularly given limitations (e.g., toxicity, suboptimal specificity) associated with current agents like 5-Aza-CdR [27, 28]. Despite the broad application of reporter gene systems in drug discovery, no HTS platform has been reported for identifying DNMT inhibitors against RCC [29–31]. Our ESRP1 promoter-driven reporter system directly addressed this gap, providing a functional cellular model to study ESRP1 promoter demethylation and significant potential for HTS of compounds targeting DNMT-mediated ESRP1 silencing in RCC. However, several limitations should be noted. First, the reporter system was developed and validated solely in the A498 RCC cell line; its performance in other RCC models or primary tumor cells remains to be determined. Second, the system is intrinsically specific to the ESRP1 promoter, limiting its direct applicability for screening inhibitors targeting other methylation-silenced genes without further validation. Third, although this study demonstrated that 5-Aza-CdR enhances ESRP1 expression in RCC cells through promoter demethylation, and an inverse correlation was observed between promoter methylation levels and both bioluminescence signal intensity and luciferase activity, the data do not exclude the possibility of indirect effects. Finally, while the system was designed for HTS, rigorous optimization and validation are still required to confirm its robustness and suitability for large-scale campaigns. Despite these limitations, this study provides a foundation for future efforts to exploit ESRP1 reactivation therapeutically and offers a specialized tool to accelerate the discovery of novel DNMT inhibitors for RCC.

## Conclusions

In conclusion, our results demonstrate that in RCC cells, ESRP1 promoter hypermethylation is associated with its transcriptional silencing; restoring ESRP1 expression induces cell cycle G1-arrest and inhibits RCC cell proliferation through downregulation of cyclin A2. Furthermore, the ESRP1-P-Luc2 reporter system represents a valuable tool for monitoring DNMT inhibitor responses at both cellular and organismal levels, providing a platform for high-throughput screening of potential anticancer drugs.

## Supplementary Information

Below is the link to the electronic supplementary material.

Supplementary Material 1

## Data Availability

The datasets analysed during the current study are available from the corresponding author on reasonable request.

## References

[1] Esteller M, Dawson MA, Kadoch C, Rassool FV, Jones PA, Baylin SB. The epigenetic hallmarks of cancer. Cancer Discov. 2024;14(10):1783–809. 39363741 10.1158/2159-8290.CD-24-0296

[2] Lee AV, Nestler KA, Chiappinelli KB. Therapeutic targeting of DNA methylation alterations in cancer. Pharmacol Ther. 2024;258:108640. 38570075 10.1016/j.pharmthera.2024.108640

[3] Mabe NW, Perry JA, Malone CF, Stegmaier K. Pharmacological targeting of the cancer epigenome. Nat Cancer. 2024;5(6):844–65. 38937652 10.1038/s43018-024-00777-2 PMC11936478

[4] Soto-Palma C, Niedernhofer LJ, Faulk CD, Dong X. Epigenetics, DNA damage, and aging. J Clin Invest. 2022;132(16):e158446. 35968782 10.1172/JCI158446 PMC9374376

[5] Kulis M, Esteller M. DNA methylation and cancer. Adv Genet. 2010;70:27–56. 20920744 10.1016/B978-0-12-380866-0.60002-2

[6] Ma L, Li C, Yin H, Huang J, Yu S, Zhao J, Tang Y. The mechanism of DNA methylation and MiRNA in breast cancer. Int J Mol Sci. 2023;24(11):9360. 37298314 10.3390/ijms24119360 PMC10253858

[7] Maiuri AR, Savant SS, Podicheti R, Rusch DB, O’Hagan HM. DNA methyltransferase Inhibition reduces inflammation-induced colon tumorigenesis. Epigenetics. 2019;14(10):1209–23. 31240997 10.1080/15592294.2019.1634986 PMC6791707

[8] Liang R, Li X, Li W, Zhu X, Li C. DNA methylation in lung cancer patients: opening a window of life under precision medicine. Biomed Pharmacother. 2021;144:112202. 34654591 10.1016/j.biopha.2021.112202

[9] Laranjeira ABA, Hollingshead MG, Nguyen D, Kinders RJ, Doroshow JH, Yang SX. DNA damage, demethylation and anticancer activity of DNA methyltransferase (DNMT) inhibitors. Sci Rep. 2023;13(1):5964. 37045940 10.1038/s41598-023-32509-4 PMC10097729

[10] Jones PA, Ohtani H, Chakravarthy A, De Carvalho DD. Epigenetic therapy in immune-oncology. Nat Rev Cancer. 2019;19(3):151–61. 30723290 10.1038/s41568-019-0109-9

[11] Lainšček D, Golob-Urbanc A, Orehek S. In vivo bioluminescence and fluorescence imaging: optical tool for cancer research. Methods Mol Biol. 2024;2773:105–23. 38236541 10.1007/978-1-0716-3714-2_11

[12] Relouw S, Dugbartey GJ, Sener A. Non-invasive imaging modalities in intravesical murine models of bladder cancer. Cancers (Basel). 2023;15(8):2381. 37190309 10.3390/cancers15082381 PMC10137013

[13] Chen ZH, Zhao RJ, Li RH, Guo CP, Zhang GJ. Bioluminescence imaging of DNA synthetic phase of cell cycle in living animals. PLoS ONE. 2013;8(11):e53291. 23301056 10.1371/journal.pone.0053291 PMC3536746

[14] Lee J, Pang K, Kim J, Hong E, Lee J, Cho HJ. ESRP1-regulated isoform switching of LRRFIP2 determines metastasis of gastric cancer. Nat Commun. 2022;13(1):6274. 36307405 10.1038/s41467-022-33786-9 PMC9616898

[15] Ala U, Manco M, Mandili G, Tolosano E, Novelli F, Provero P. Proteomics-based evidence for a pro-oncogenic role of ESRP1 in human colorectal cancer cells. Int J Mol Sci. 2020;21(2):575. 31963158 10.3390/ijms21020575 PMC7014300

[16] Harvey SE, Xu Y, Lin X, Gao XD, Qiu Y, Ahn J. Coregulation of alternative splicing by HnRNPM and ESRP1 during EMT. RNA. 2018;24(11):1326–38. 30042172 10.1261/rna.066712.118 PMC6140460

[17] Göttgens EL, Span PN, Zegers MM. Roles and regulation of epithelial splicing regulatory proteins 1 and 2 in epithelial-mesenchymal transition. Int Rev Cell Mol Biol. 2016;327:163–94. 27692175 10.1016/bs.ircmb.2016.06.003

[18] Ueda J, Matsuda Y, Yamahatsu K, Uchida E, Naito Z, Korc M. Epithelial splicing regulatory protein 1 is a favorable prognostic factor in pancreatic cancer that attenuates pancreatic metastases. Oncogene. 2014;33(45):4485–95. 24077287 10.1038/onc.2013.392 PMC4041859

[19] Pan Y, Zhao Y, Li L, Xie Y, Zou Q. MiR-337-3p suppresses migration and invasion of breast cancer cells by downregulating ESRP1. Acta Histochem. 2021;123:151777. 34481218 10.1016/j.acthis.2021.151777

[20] Chen ZH, Jing YJ, Yu JB, Jin ZS, Li Z, He TT, Su XZ. ESRP1 induces cervical cancer cell G1-phase arrest via regulating Cyclin A2 mRNA stability. I J Mol Sci. 2019;20:3705. 10.3390/ijms20153705 PMC6695732 31362365

[21] Pagano M, Pepperkok R, Verde F, Ansorge W, Draetta G. Cyclin A is required at two points in the human cell cycle. EMBO J. 1992;11:961–71. 1312467 10.1002/j.1460-2075.1992.tb05135.x PMC556537

[22] Yang Q, Yoshimura G, Mori I, Sakurai T, Kakudo K. Chromosome 3p and breast cancer. J Hum Genet. 2002;47(9):453–9. 12202982 10.1007/s100380200064

[23] Calvanese V, Horrillo A, Hmadcha A, Suarez-Alvarez B, Fernandez AF, Lara E, Casado S, Menendez P, Bueno C, Garcia-Castro J, Rubio R, Lapunzina P, Alaminos M, Borghese L, Terstegge S, Harrison NJ, Moore HD, Brüstle O, Lopez-Larrea C, Andrews PW, Soria B, Esteller M, Fraga MF. Cancer genes hypermethylated in human embryonic stem cells. PLoS ONE. 2008;3(9):e3294. 18820729 10.1371/journal.pone.0003294 PMC2546447

[24] Jeong HM, Han J, Lee SH, Park HJ, Lee HJ, Choi JS, Lee YM, Choi YL, Shin YK, Kwon MJ. ESRP1 is overexpressed in ovarian cancer and promotes switching from mesenchymal to epithelial phenotype in ovarian cancer cells. Oncogenesis. 2017;6(10):e389. 28991261 10.1038/oncsis.2017.87 PMC5668885

[25] Kondratov AG, Kvasha SM, Stoliar LA, Romanenko AM, Zgonnyk YM, Gordiyuk VV, Kashuba EV, Rynditch AV, Zabarovsky ER, Kashuba VI. Alterations of the WNT7A gene in clear cell renal cell carcinomas. PLoS ONE. 2012;7(10):e47012. 23056560 10.1371/journal.pone.0047012 PMC3466251

[26] Majid S, Dar AA, Ahmad AE, Hirata H, Kawakami K, Shahryari V, Saini S, Tanaka Y, Dahiya AV, Khatri G, Dahiya R. BTG3 tumor suppressor gene promoter demethylation, histone modification and cell cycle arrest by genistein in renal cancer. Carcinogenesis. 2009;30(4):662–70. 19221000 10.1093/carcin/bgp042 PMC2664457

[27] Mehdipour P, Chen R, De Carvalho DD. The next generation of DNMT inhibitors. Nat Cancer. 2021;2:1000–1. 35121882 10.1038/s43018-021-00271-z

[28] Ye C, Jiang N, Zheng J, Zhang S, Zhang J, Zhou J. Epigenetic therapy: research progress of decitabine in the treatment of solid tumors. Biochim Biophys Acta Rev Cancer. 2024;1879:189066. 38163523 10.1016/j.bbcan.2023.189066

[29] Tang N, Li L, Xie F, Lu Y, Zuo Z, Shan H, Zhang Q, Zhang L. A living cell-based fluorescent reporter for high-throughput screening of anti-tumor drugs. J Pharm Anal. 2021;11(6):808–14. 35028187 10.1016/j.jpha.2021.04.001 PMC8740116

[30] Li Y, Zhou W, Meng X, Murray SD, Li L, Fronk A, Lazaro-Camp VJ, Wen KK, Wu M, Dupuy A, Leslie KK, Yang S. Utilizing an endogenous progesterone receptor reporter gene for drug screening and mechanistic study in endometrial cancer. Cancers (Basel). 2022;14(19):4883. 36230806 10.3390/cancers14194883 PMC9561963

[31] Kelly T, Yang X. Application of fluorescence- and bioluminescence-based biosensors in cancer drug discovery. Biosens (Basel). 2024;14(12):570. 10.3390/bios14120570 PMC11674898 39727835

