# Supplementary material for: The ESRP1 promoter reporter can function as an in vivo sensor of DNA methyltransferase inhibition
Source: BMC Biotechnol. 2025 Aug 27;25:90. doi: 10.1186/s12896-025-01031-y (PMC12382134; doi:10.1186/s12896-025-01031-y)
Supplement: Supplementary file 1 — Supplementary Material 1 [file 12896_2025_1031_MOESM1_ESM.pdf]

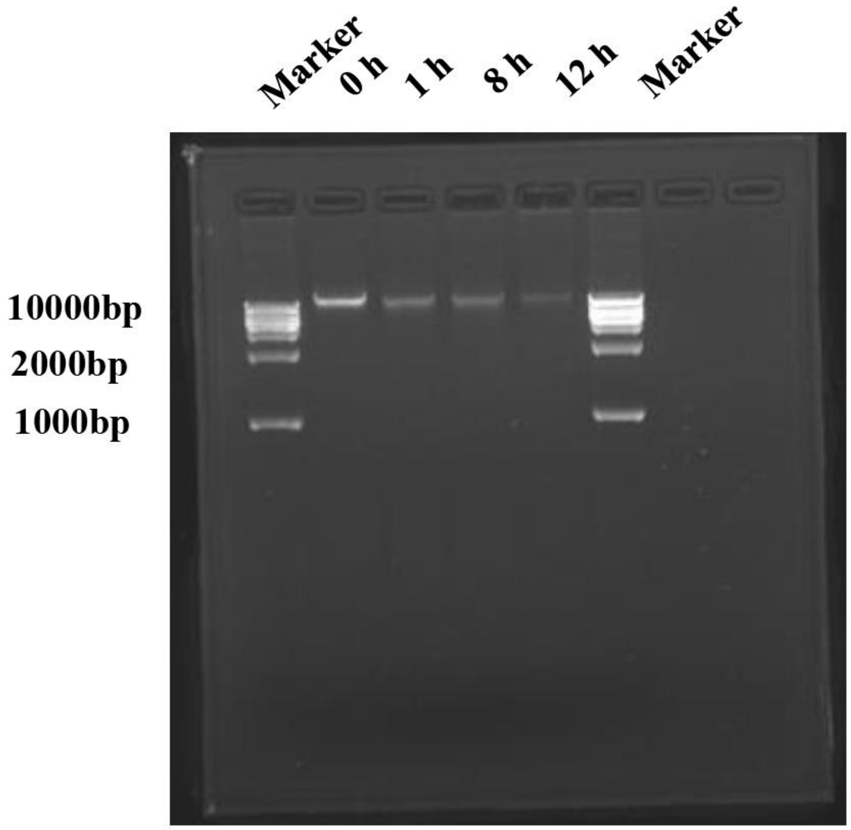

Figure 1I. Genomic DNA from HEK293 cells was digested with HhaI for 0, 1, 8, and 12 hours, followed by analysis using agarose gel electrophoresis.

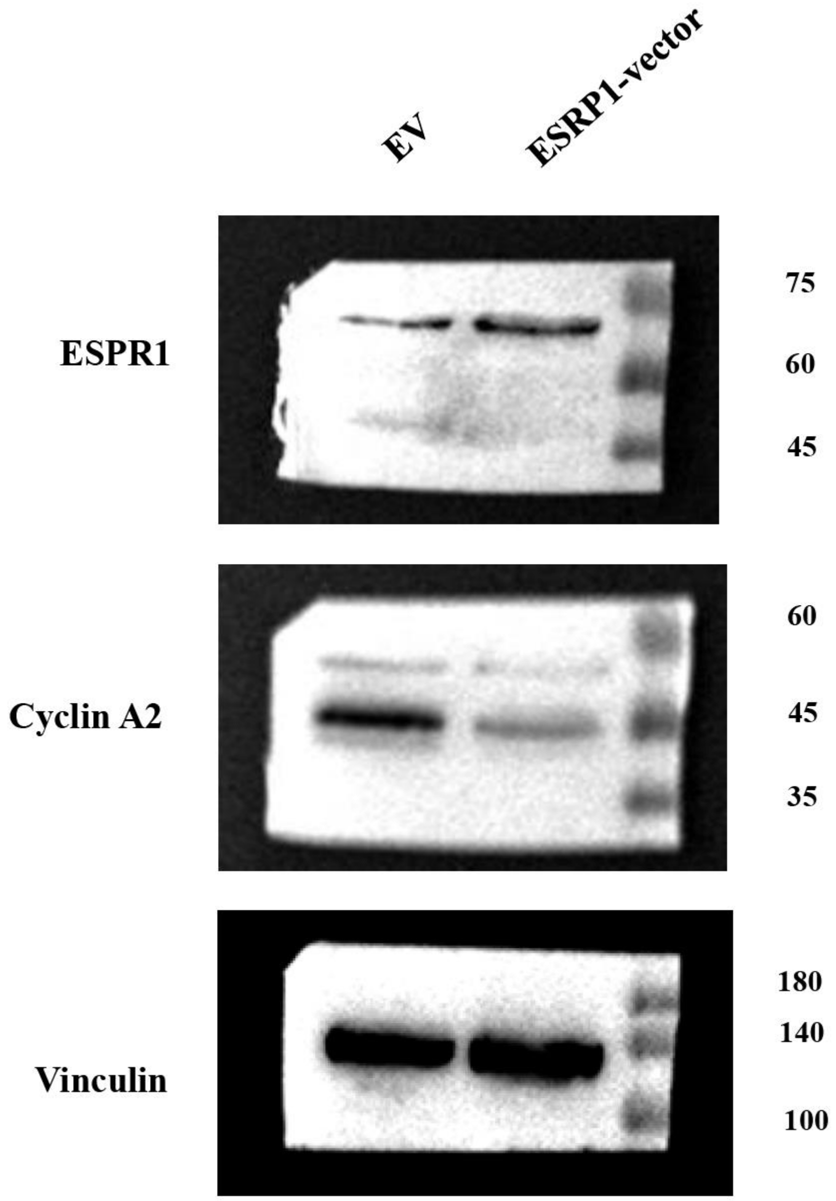

Figure 2E. After transfection with ESRP1 vector or empty vector (EV) for 48 hours, the expression of ESRP1 and Cyclin A2 in A498 cells was detected by Western blotting.

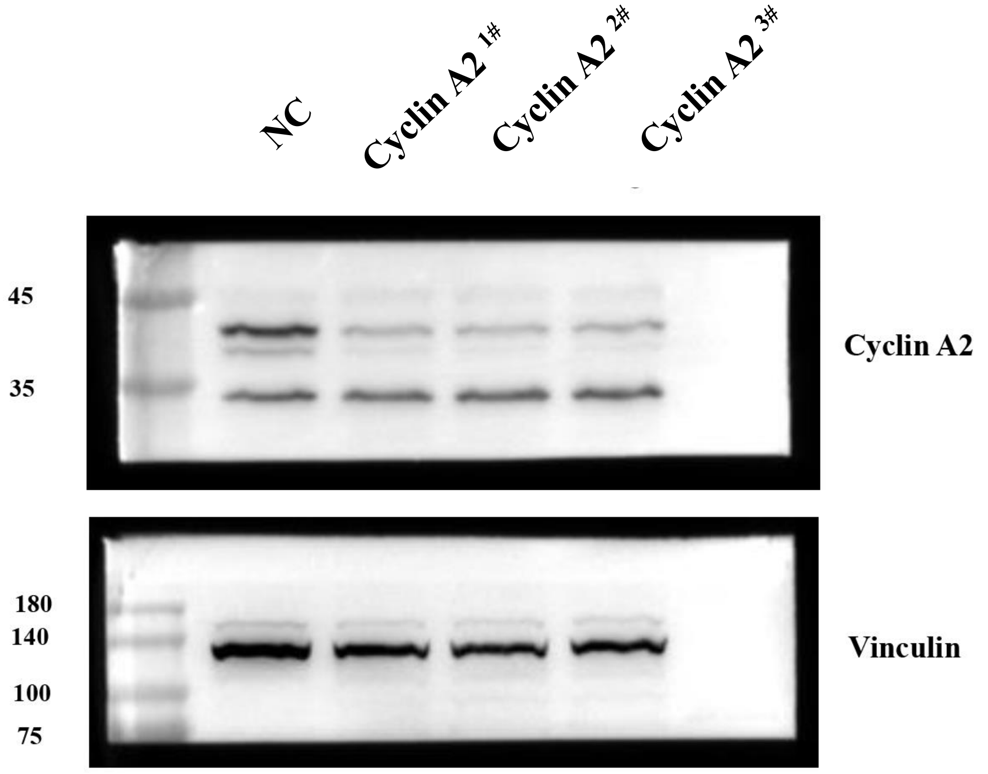

Figure 2G. At 48 h after transfection with NC or 50 nM Cyclin A2 siRNA (Cyclin A2 siRNA [1#], Cyclin A2 siRNA [2#], and Cyclin A2 siRNA [3#]), Cyclin A2 levels were measured in A498 cells by Western blotting.

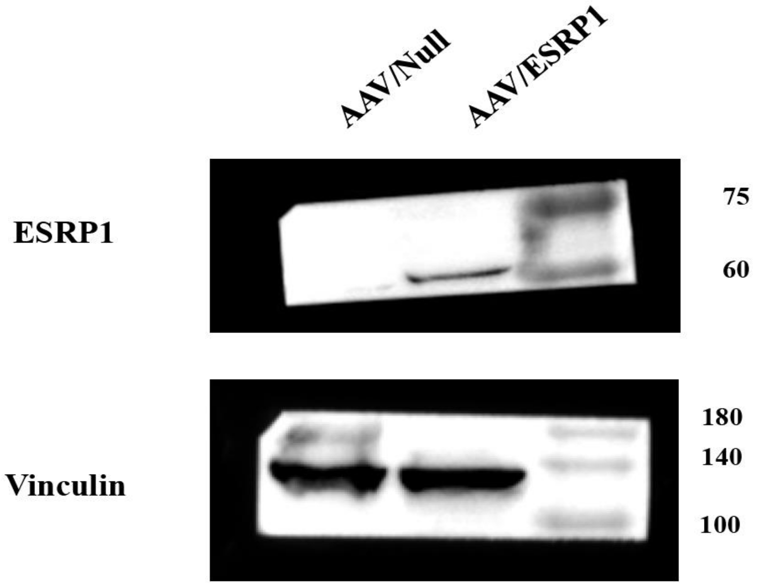

Figure 2K. The expression of ESRP1 in tumor tissues of nude mice injected with AAV/ESRP1 or AAV/Null was detected by Western blotting.

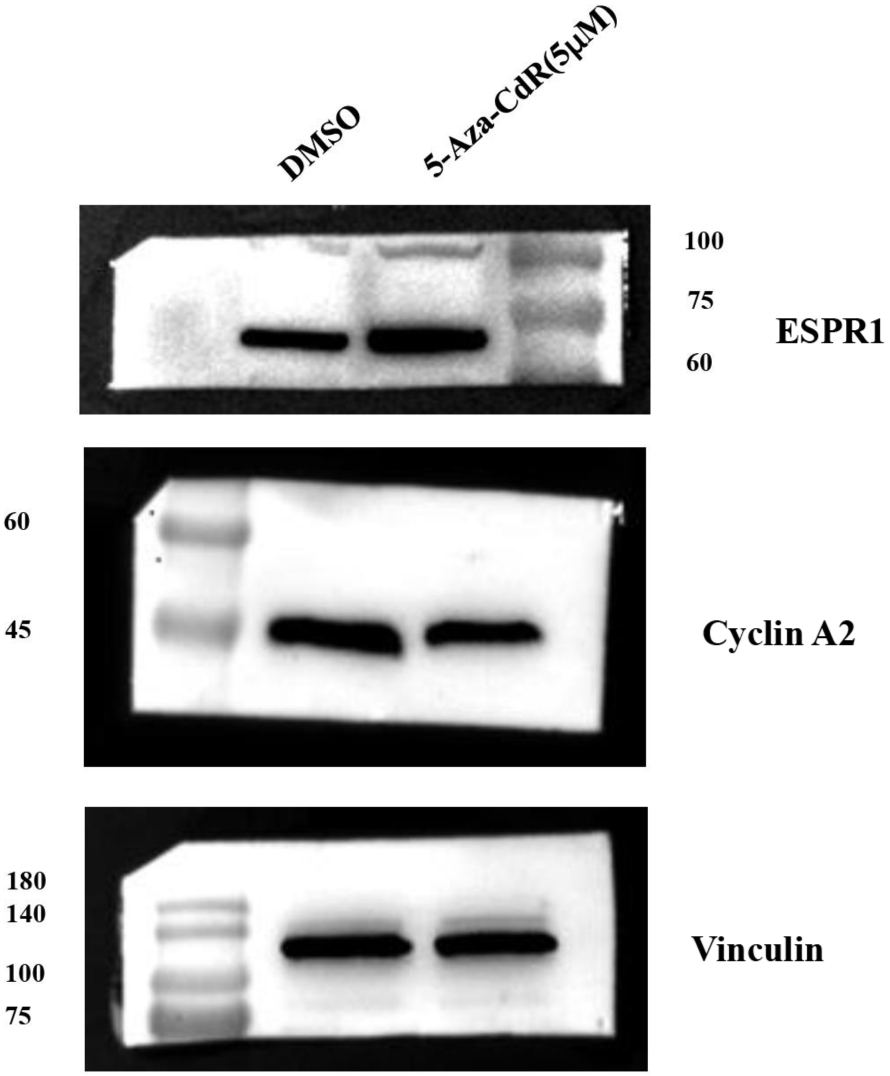

Figure 3D. After treatment with 5 μM 5-Aza-CdR for 48 h, the expression of ESRP1 and Cyclin A2 in A498 cells was detected by Western blotting.

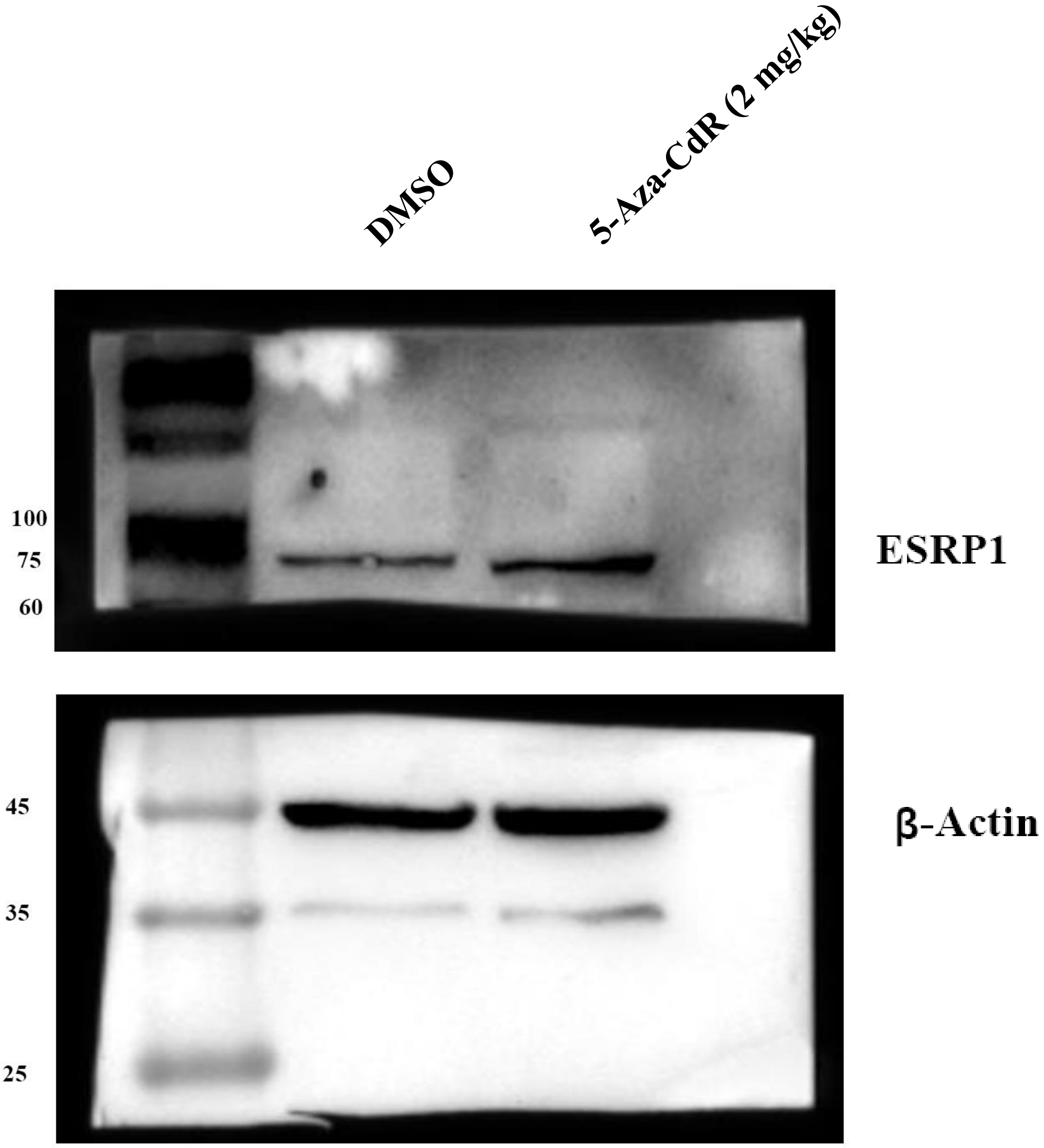

Figure 5G. Nude mice were administered DMSO or 5-Aza-CdR (2 mg/kg) via intraperitoneal injection. Tumor tissues were harvested 48 hours later, and ESRP1 protein expression was determined by Western blotting.
